# Improved Efficiency of Silicon Nanoholes/Gold Nanoparticles/Organic Hybrid Solar Cells via Localized Surface Plasmon Resonance

**DOI:** 10.1186/s11671-016-1374-0

**Published:** 2016-03-22

**Authors:** Ronghua Lu, Ling Xu, Zhaoyun Ge, Rui Li, Jun Xu, Linwei Yu, Kunji Chen

**Affiliations:** National Laboratory of Solid State and Microstructures School of Electronic Science and Engineering, Nanjing University, Nanjing, 210093 People’s Republic of China

**Keywords:** AgNPS/SiNHS nanocomposite films, Surface passivation, AuNPS, LSPR

## Abstract

Silicon is the most widely used material for solar cells due to its abundance, non-toxicity, reliability, and mature fabrication process. In this paper, we fabricated silicon nanoholes (SiNHS)/gold nanoparticles (AuNPS)/organic hybrid solar cells and investigated their spectral and opto-electron conversion properties. SiNHS nanocomposite films were fabricated by metal-assisted electroless etching (EE) method. Then, we modified the surface of the nanocomposite films by exposing the samples in the air. After that, polymer poly(3,4-ethylenedioxythiophene):poly (styrenesulfonate) (PEDOT:PSS) blended with AuNPS were spin-coated on the surface of the SiNHS nanocomposite films as a hole-transporting layer. The external quantum efficiency (EQE) values of the solar cells with AuNPS are higher than that of the samples without AuNPS in the spectral region of 600–1000 nm, which were essential to achieve high performance photovoltaic cells. The power conversion efficiency (PCE) of the solar cells incorporating AuNPS exhibited an enhancement of 27 %, compared with that of the solar cells without AuNPS. We thought that the improved efficiency were attributed to localized surface plasmon resonance (LSPR) triggered by gold nanoparticles in SiNHS nanocomposite films.

## Background

A solar cell is a promising form of solar energy because of its permanence and cleanness. Polymer poly(3,4-ethylenedioxythiophene):poly (styrenesulfonate) (PEDOT:PSS) hybrid Schottky solar cells have attracted great attention in a variety of solar cells because this kind of solar cells has shown the potential as low-cost and high-efficiency photovoltaic devices and can be fabricated simply [1, 2]. More importantly, PEDOT:PSS/Si solar cells have achieved a power conversion efficiency (PCE) of over 13 % and achieved a record efficiency of 13.8 % by using MoO_3_ film as an antireflection and inversion induced layer [3, 4]. In order to utilize the solar energy as far as possible, it is very necessary to improve the light absorption and power conversion efficiency of solar cells in a wide spectral range. Nanomaterials and nanostructures such as silicon nanowires (SiNWS) and silicon nanoholes (SiNHS) have been extensively investigated for hybrid solar cells due to their unique photoelectric properties and technical compatibility with Si-based semiconductor technology [2, 5]. They can be easily fabricated by the metal-assisted electroless etching method at room temperature and served as an antireflection layer with good performance for solar cells [5–7]. Moreover, compared with plane silicon, they have high aspect ratios possessing significant light-trapping capability and superior charge separation capability. In addition, SiNWS might be limited in the use of devices because of the fragile mechanical nature of freestanding SiNWS. However, SiNHS with greater mechanical stability can solve this problem. It has been reported that SiNHS with excellent antireflection properties were applied to enhance the optical absorption performance in Si-based solar cells [8].

Furthermore, in order to improve the conversion efficiency of hybrid solar cells based on silicon nanostructures, some groups have paid attention to the application of the surface plasmon resonance technique [9, 10]. Localized surface plasmon resonance is an important optical near-field effect on localized surface in metal nanoparticles, which can lead to strong enhancement of the local electromagnetic field, and the fields are a benefit to the photo-induced charge separation. When the surface plasmon resonance of metal nanoparticles occurs, the light absorption of the material increases, which is important for short wavelength spectral response of solar cells based on silicon [9, 11, 12]. Therefore, the localized surface plasmon resonance (LSPR) can provide a possible way to achieve a high photoelectric conversion efficiency. However, we noted that the surface of silicon nanostructures made by wet chemistry method can produce some defects, and the existence of the defects would increase the recombination probability of carriers [13, 14]. And it is reported that interface defects play an important role in device characteristics, so some teams carried out surface modification of nanostructures to suppress the surface defects and mitigate interface carrier recombination to improve the performance of silicon/organic hybrid solar cells [14–17]. In addition, carrier recombination can also occur in the metal nanoparticles and SiNHS contact surfaces. So the ultrathin oxide layer between metal nanoparticles and SiNPS obtained by surface passivation can effectively inhibit the recombination of photo-generated charges [18].

In this work, we fabricated SiNHS/gold nanoparticles (AuNPS)/PEDOT:PSS hybrid solar cells by electroless etching (EE) method to improve the performance via reducing interface carrier recombination and enhancing the light absorption at short wavelength based on LSPR effects. Through passivating the SiNHS surface and adjusting the mass ratio of AuNPS mixed with PEDOT:PSS, substantial improvement of device performance was obtained, leading to an optimal PCE of 6.1 %.

## Methods

Silver nanoparticles (AgNPS)/SiNHS nanocomposite films were fabricated by a modified EE method at room temperature. Firstly, AgNPS prepared in aqueous solution [19] were deposited on clean n-type silicon wafers by spin-coating and then the samples were immersed into an aqueous mixture solution of 5 M HF acid and 0.2 M hydrogen peroxide (H_2_O_2_) for the formation of SiNHS. The AgNPS fell into the holes in the etching processes. When the etching processes were over, the samples were rinsed with deionized water directly and dried by the blowing of nitrogen. Secondly, the clean samples were placed in the air for 1.5 h to make the surface form SiOx–Si bonds [17]. Finally, the AgNPS/SiNHS nanocomposite films were formed by silver nanoparticles embedded in a Si nanohole matrix [20]. The geometrical features of the films including length of holes and wall thickness were controlled by a variety of parameters such as etching time, size, and distance of AgNPS.

After fabricating the AgNPS/SiNHS nanocomposite films, we began to manufacture the SiNHS/Au NPS/PEDOT:PSS hybrid solar cells. At first, AuNPS in aqueous solution were prepared [21] and mixed with PEDOT:PSS. And then the mixed solution was spin-coated on the films at 3000 rpm for 60 s to fabricate the heterojunction device structure. Subsequently, aluminum (Al) and Ag were deposited on the rear and front sides of the samples, respectively, by magnetron sputtering, which enabled the formation of ohmic contacts between electrodes and substrates.

**Fig. 1:**
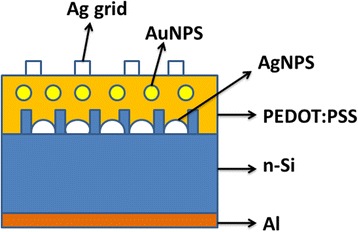
Schematic illustration of PEDOT:PSS/AuNPS/(AgNPS/SiNHS nanocompoite films) hybrid solar cells

The morphology of the samples was characterized by a scanning electron microscope (SEM) and an atomic force microscopy (AFM). The optical absorption of the cells was measured at room temperature by Shimadzu UV-3600 spectrophotometer. The illuminated *I*–*V* characteristics were measured under an AM 1.5 G at 100 mW/cm^2^ illumination, and the external quantum efficiency (EQE) spectra were obtained by the spectral response measurement system. Figure 1 depicts the schematic illustration of the hybrid solar cells based on SiNHS and Au NPS/PEDOT:PSS with silver particles.

## Results and Discussion

### Structure Characterization of SiNHS/PEDOT:PSS Nanocomposite Films

**Fig. 2:**
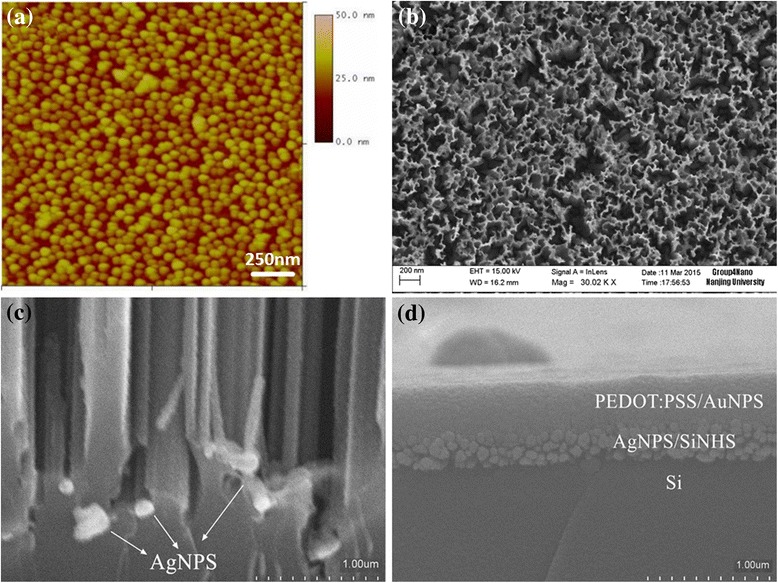
**a** The AFM image of Au nanoparticles. **b** Top-view SEM image of AgNPS/SiNHS nanocomposite films. **c** Cross section SEM image of the AgNPS/SiNHS nanocomposite films. **d** Cross section SEM image of the films coated with PEDOT:PSS/AuNPS

Figure 2a shows the AFM image of Au nanoparticles spin-coated on Si wafers (flat side). We can see that AuNPS are round, and the average diameter of the AuNPS was estimated to be 50 ± 5 nm. Top-view SEM image of AgNPS/SiNHS nanocomposite films is showed in Fig. 2b. It can be observed that the silicon nanoholes are formed and the distance between them is about 150 nm. Figure 2c shows the cross section SEM images of the SiNHS/AgNPS films. As shown in the picture, AgNPS were embedded in the SiNHS. Figure 2d exhibits the cross section SEM image of the heterojunction based on SiNHS/AgNPS coated with PEDOT:PSS/AuNPS. It can be seen that the PEDOT:PSS/AuNPS can be well filled into SiNHS and the surface of the samples is smooth.

### Performance of Solar Cells

To further understand the effect of silicon nanostructure surface passivation on the overall performance of the solar cell, we introduced a SiOx–Si bond at the organic-silicon interface of hybrid heterojunction solar cells based on SiNHS [17]. The result of PCE shows that the highest PCE reaches a record of 5.5 % in comparison to 4.8 % from the reference counterpart without the SiOx–Si bond.

**Fig. 3:**
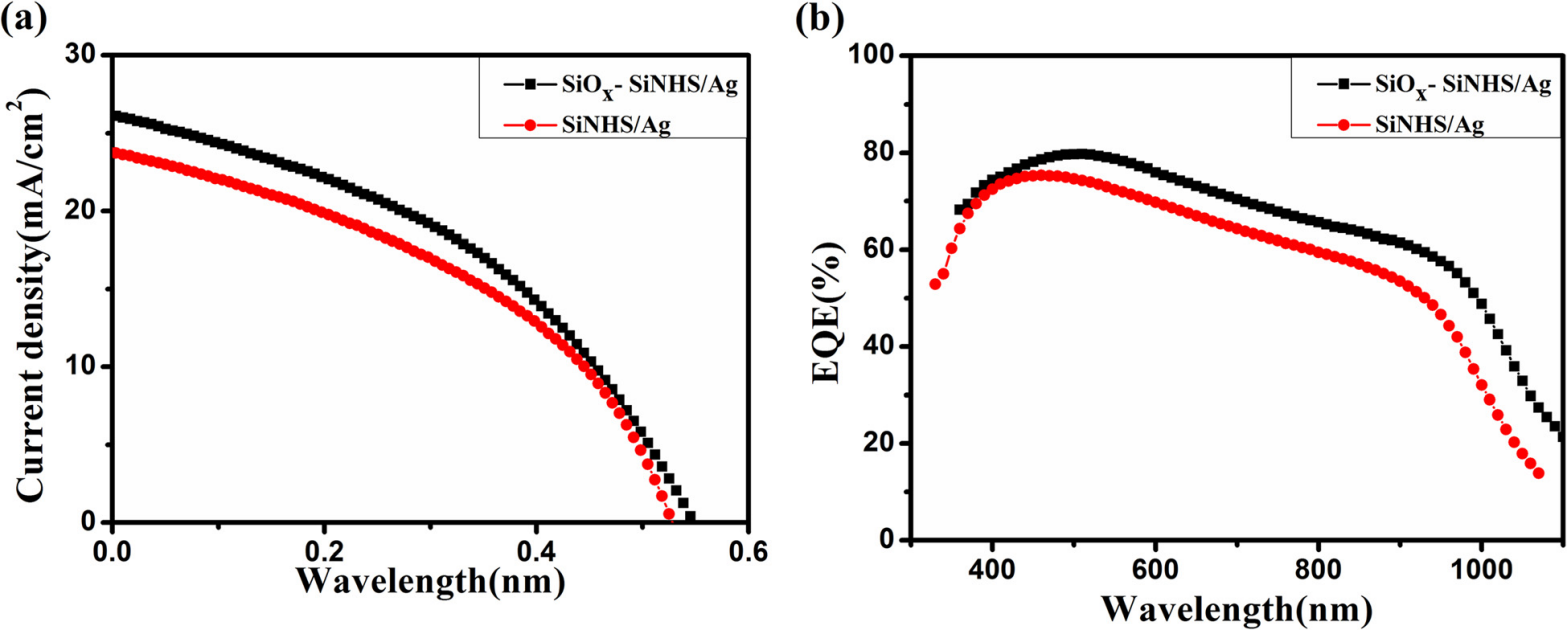
**a** Current density–voltage (*J*–*V*) characteristics of hybrid solar cells with and without SiOx–Si bond. **b** EQE spectra of hybrid solar cells with and without SiOx–Si bond

In addition, we measured the current density–voltage (*J*–*V*) characteristics and EQE of these solar cells based on the surface modification of the films (SiOx–SiNH/Ag) and the films without surface modification (SiNH/Ag), respectively (shown in Fig. 3). The *J*–*V* characteristics were measured under simulated AM 1.5 G at 100 mW/cm^2^ illumination. The measured photovoltaic parameters of short circuit current density (*J*_sc_), open circuit voltage (*V*_oc_), fill factor (FF), and power conversion efficiency (*η*) are listed in Table 1. As shown in Fig. 3 and Table 1, the short circuit current and EQE of the devices based on SiOx–SiNHS/AgNPS nanocomposite films were significantly increased compared to those of the devices based on SiNHS/AgNPS nanocomposite films. This is mainly because the surface modification of the SiNHS/AgNPS nanocomposite film can reduce defects on the surface and inhibit the carrier surface recombination [4, 14, 15]. The ultrathin oxides layer obtained by surface passivation can also inhibit the recombination of photo-generated charges in the metal nanoparticles and SiNPS contact surfaces [18]. In this way, the fill factor was increased from 39.4 to 41.6 %, and the conversion efficiency had an enhancement up to 25 %. We can see that the EQE value of the passivated sample increased significantly in the most part of the wavelength range. The existence of the defects can make the electrons and holes recombine in the cells. In this work, SiNHS/AgNPS nanocomposite films were further modified with the SiOx–Si bond by exposing the films in the air to suppress the interface defects. As a result, the overall performance of the solar cells was improved [22]. The surface passivation of the nanocomposite films has a great impact on the performance of solar cells, and we can use this method to improve the PCE of organic-silicon substrate hybrid heterojunction solar cells.

**Table 1:** Device performances of hybrid solar cells with and without SiOx–Si bond

Devices	*V*_oc_ (V)	*J*_sc_ (mA/cm^2^)	FF (%)	PCE (%)
SiNHS/Ag	0.527	23.6	39.4	4.8
SiOx–SiNHS/Ag	0.538	26.1	41.6	5.5

Moreover, the low light absorption efficiency of organic-silicon substrate will affect the PCE of hybrid solar cells. So far, the application of the LSPR induced by metal nanoparticles has received extensive attention in the field of solar cells [9, 10]. Moreover, some researchers introduced metal nanoparticles into a PEDOT:PSS buffer layer to achieve light scattering, and they also blended metal nanoparticles with different LSPR wavelengths into a PEDOT:PSS buffer layer to enhance the light-trapping efficiency of hybrid heterojunction solar cells [9, 10]. Therefore, to improve the light absorption efficiency and boost the PCE of the solar cells, we introduce the AuNPS into the organic materials on the basis of passivated nanocomposite films.

In order to investigate the effect of LSPR on the overall performance of the organic-silicon substructure solar cell, we studied the light absorption spectrum of the colloidal AgNPS, AuNPS, and AuNPS/SiNHS nanocomposite films. Firstly, we used the Shimadzu UV-3600 spectrophotometer to measure the transmission (*T*) and reflection (*R*) spectra of the samples. The absorbance (*A*) spectrum of the samples were calculated by the following formula:

$$ A=1-T-R. $$

**Fig. 4:**
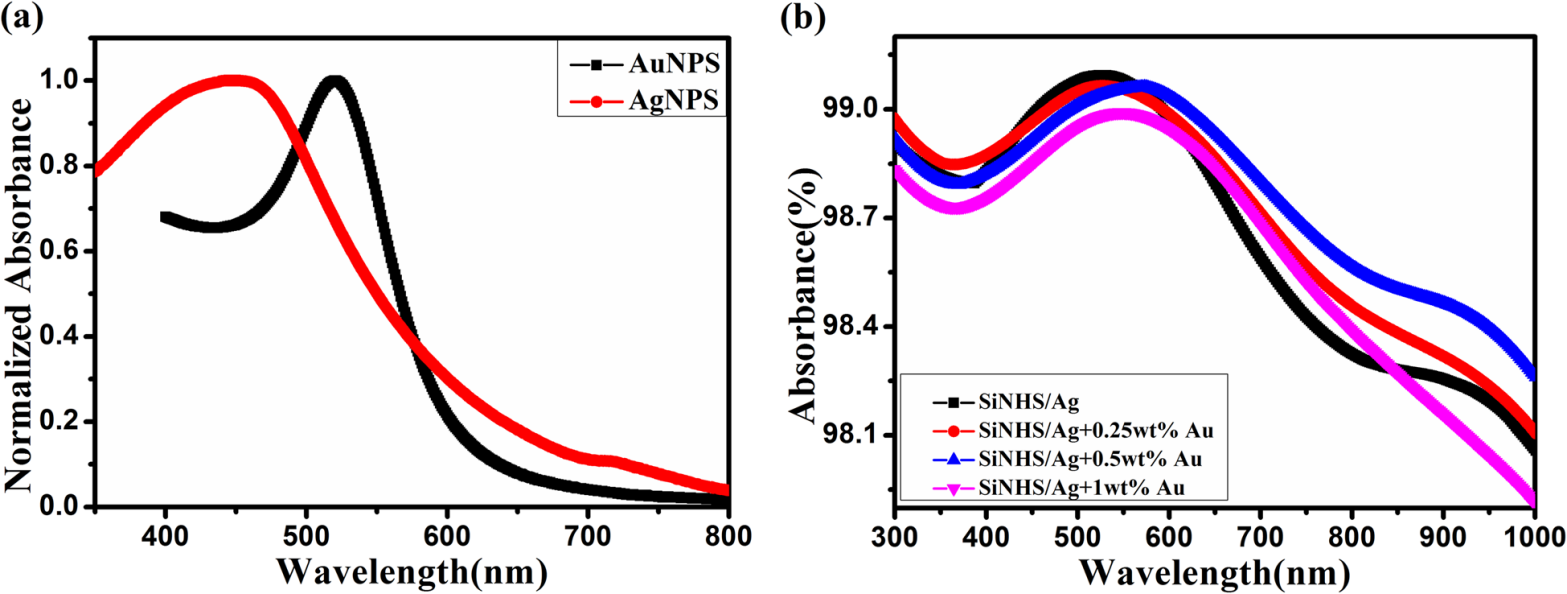
**a** Optical absorption spectra of colloidal AuNPS dispersed in deionized water. **b** Optical absorption spectra of the PEDOT:PSS/AuNPS/(AgNPS/SiNHS) nanocomposite films in different etching time and the films without AgNPS

For the purpose of making better use of gold nanoparticles to increase the light absorption of solar cells, we adopted the spherical gold particles, in which the diameter is 50 nm [23]. Figure 4a shows the absorption spectra of the AuNPS and AgNPS dispersed in deionized water. We observed that the absorption peak of the AgNPS and AuNPS is near 480 and 520 nm, respectively. We attributed the light absorption peak to the plasmon resonance absorption of the nanoparticles. Figure 4b depicts the absorption spectra of AgNPS/SiNHS nanocomposite films with different mass ratios of AuNPS mixed with PEDOT:PSS. It should be noted that the peak of absorption was redshifted to around 550 nm due to charge transfer between the proximal nanoparticles and between nanoparticle and silicon substrate. This can cause energy loss due to collisions between charge (electrons or holes) and lattice atoms, resulting in the redshift of the absorption peak [24–26]. Furthermore, with the increase of mass ratio of AuNPS, the light absorption of the devices increased in the wavelength beyond 600 nm. This is mainly due to light scattering leading to an increment in the light absorption of films. The increased light absorption of the samples in long wavelength range reached the maximum at the mass ratio of AuNPS 0.5 wt%, and an enhancement of 16.1 % in total absorption throughout the wavelength range of 300–1000 nm is obtained by integrating method.

The LSPR effect caused by AuNPS and AgNPS can extend the absorption peak of nanocomposite films, so, with the introduction of AuNPS, the absorption of the solar cells would be increased. AuNPS can also enhance the light scattering of the devices [9]. With the increment of mass ratio of AuNPS, light absorption was enhanced and reached the maximum at 0.5 wt%. However, the light absorption of the devices began to weaken when the AuNPS continued to increase. This is mainly because too many particles on the surface of SiNHS will hinder the absorption of the nanocomposite films. Therefore, we think the best mass ratio of AuNPS is 0.5 wt%.

To research the effect of metal nanoparticles to the performance of the PEDOT:PSS/AuNPS/(AgNPS/SiNHS) nanocompoite films hybrid solar cells, we measured the current density–voltage characteristics under simulated AM 1.5 G at 100 mW/cm^2^ illumination and EQE of these solar cells. Their photovoltaic parameters *V*_oc_, *J*_sc_, FF, and PCE are summarized in Table 2.

**Table 2:** Device performances of hybrid solar cells with AuNPS or AgNPS and without

Devices	*V*_oc_ (V)	*J*_sc_ (mA/cm^2^)	FF (%)	PCE (%)
SiOx–SiNHS/Ag + Au	0.531	27.3	42.1	6.1
SiOx–SiNHS/Ag	0.541	25.5	40.8	5.5
SiOx–SiNHS	0.501	20.8	22.9	2.4

**Fig. 5:**
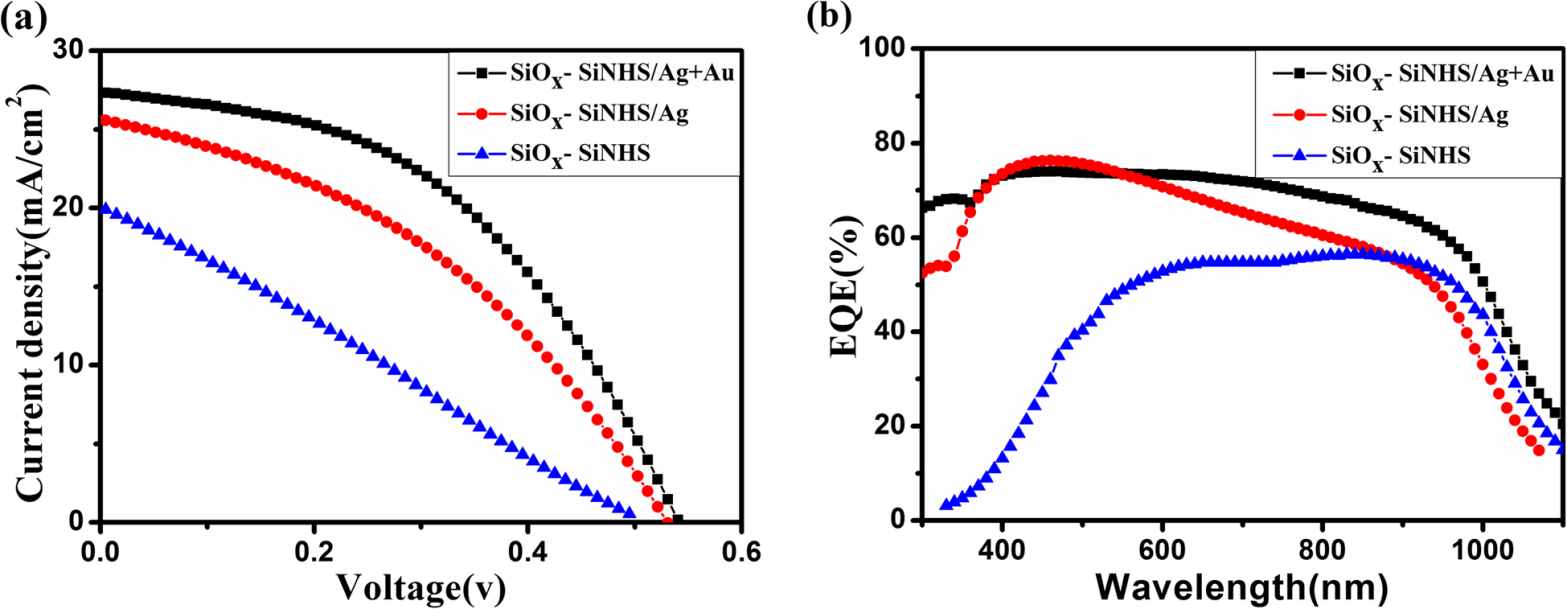
**a** Current density–voltage (*J*–*V*) characteristics of hybrid solar cells with and without AuNPS or AgNPS. **b** EQE spectra of hybrid solar cells with and without AuNPS or AgNPS

As seen in Fig. 5 and Table 2, the performances of the devices with metal nanoparticles were significantly improved compared to those of the devices without nanoparticles, which might result from the high light harvesting efficiency of the devices. We attributed the high light harvesting efficiency to the effect of plasmon resonance induced by metal nanoparticles.

From Fig. 5a, we also observed that the short circuit current density of the samples with AuNPS were higher than that of the devices without AuNPS. As shown in Table 2, the short circuit current of the devices increased by 7 %, and the power conversion efficiency of the solar cells increased from 5.5 to 6.1 %. The AuNPS and AgNPS have different LSPR peak positions (as seen in Fig. 4a), thus enhancing the light absorption of the devices, so the solar cells parameters were boosted markedly [9]. Besides, the EQE, another important parameter of the solar cells, is shown in Fig. 5b. We noted that the EQE value of the devices with AuNPS increased observably compared to the devices without AuNPS throughout the wavelength after 600 nm, and that is closely related to the absorption spectrum of the solar cells (shown in Fig. 4b). Therefore, the introduction of the AuNPS can increase the light scattering in the devices, which will also improve the light absorption efficiency. Moreover, the LSPR effect caused by metal nanoparticles can enhance the local electromagnetic field around the particles, and the field is a benefit to the photo-induced charge separation [27]. With AuNPS, the ability of the devices to capture light was enhanced and the performance parameters of the solar cells were improved significantly.

Based on the results of experiments, we can reduce the surface carrier recombination and improve the performance of solar cells after passivating the surface of silicon nanostructure films. In addition, the LSPR effect based on nanoparticles leads to enhancement of local electromagnetic fields and optical absorption. And the light scattering also increased, which will lengthen the optical path in the SiNHS and improve the light absorption efficiency. So, these two processes lead to the improvement of the properties of SiNHS/AuNPS/organic hybrid solar cells.

## Conclusions

In summary, hybrid solar cells of PEDOT:PSS/(AgNPS/SiNHS) nanocomposite film with gold nanoparticles were fabricated by EE method. The performances of solar cells were enhanced after modifying the surface of the AgNPS/SiNHS nanocomposite films, and it was found that the optical absorption spectra of the films with metal nanoparticles showed the enhancement within the wavelength range from 600 to 850 nm. The result of light absorption measurements showed that the optimal value for AuNPS mass ratio is 0.5 wt%. Through passivating the surface of the films and blending the AuNPS into PEDOT:PSS buffer layers, we enhanced the PCE from 4.8 to 6.1 %, with a high enhancement factor of 27 %. Moreover, after introducing the AuNPS into the devices, the EQE value increased significantly in a spectral range of 600–1000 nm, and the *J*_sc_ and FF also increased obviously. We think these were mainly attributed to LSPR effects and increment of light scattering of AgNPS. We believe that such approaches of modifying the surface and blending the AuNPS into PEDOT:PSS buffer layers would be a promising candidate for solar cells application.

## Abbreviations

AgNPS: silver nanoparticles
AuNPS: gold nanoparticles
EQE: external quantum efficiency
LSPR: localized surface plasmon resonance
PCE: power conversion efficiency
PEDOT:PSS: polymer poly(3,4-ethylenedioxythiophene):poly (styrenesulfonate)
SiNHS: Si nanoholes
SiNWS: silicon nanowires
